# Decision impact and feasibility of different ASCO-recommended biomarkers in early breast cancer: Prospective comparison of molecular marker EndoPredict and protein marker uPA/PAI-1

**DOI:** 10.1371/journal.pone.0183917

**Published:** 2017-09-06

**Authors:** Johannes Ettl, Evelyn Klein, Alexander Hapfelmeier, Kirsten Grosse Lackmann, Stefan Paepke, Christoph Petry, Katja Specht, Laura Wolff, Heinz Höfler, Marion Kiechle

**Affiliations:** 1 Klinik und Poliklinik für Frauenheilkunde, Klinikum rechts der Isar, Technische Universität München, Munich, Germany; 2 Institute of Medical Statistics and Epidemiology, Klinikum rechts der Isar, Technische Universität München, Munich, Germany; 3 Sividon Diagnostics GmbH, Cologne, Germany; 4 Institute of Pathology, Klinikum rechts der Isar, Technische Universität München, Munich, Germany; University of North Carolina at Chapel Hill School of Medicine, UNITED STATES

## Abstract

**Background:**

Adjuvant therapy decisions in early breast cancer are based on accurate risk assessment. Urokinase plasminogen activator (uPA) and plaminogen activator inhibitor-1 (PAI-1) have been the first biomarkers in hormone receptor (HR) positive breast cancer to reach highest level of evidence. The EndoPredict test (EPclin) combines gene expression information with nodal status and tumor size. The aim of this prospective study was to compare uPA/PAI-1 and EPclin as prognostic biomarkers with regard to feasibility, risk stratification and impact on adjuvant therapy recommendation.

**Materials and method:**

395 patients with HR positive, HER2 negative, intermediate risk breast cancer were enrolled. Relations and concordance of histologic grading as well as EPclin and uPA/PAI-1 values were assessed by Spearman’s rank correlation coefficient and Cohen’s Kappa. To compare decision impact of EPclin and uPA/PAI-1 three independent case discussions were held: One with known uPA/PAI-1 and EPclin results, one blinded to EPclin alone and another one blinded to both EPclin and uPA/PAI-1.

**Results:**

EPclin could be determined in all 395 (100%), uPA/PAI-1 in 190 (48%) of the tumor samples. EPclin allocated 250 patients (63%) to the low-risk group and 145 patients (37%) to the high-risk group, whereas uPA/PAI-1 allocated 88 patients (46%) to the low-risk group and 102 patients (54%) to the high-risk group. In 59% of cases, both tests showed concordant results. EPclin resulted more frequently in a change of therapy recommendation than the uPA/PAI-1 test (46% vs 24%). Recommendation of adjuvant chemotherapy (CTX) was abandoned twice as often by EPclin (45%) compared to uPA/PAI-1 (22%).

**Conclusion:**

In this first prospective comparison of EPclin and uPA/PAI-1 we found, that EPclin is superior to uPA/PAI-1 with respect to feasibility and decision impact. This leads to substantial avoidance of adjuvant CTX in endocrine-sensitive, HER2-negative breast cancer. Data collection for patients´ clinical outcome is ongoing.

## Introduction

Patients diagnosed with early breast cancer are faced with life-long risk of distant metastases. Nevertheless, along with intensified early detection and optimized adjuvant systemic therapies, an improvement of disease-free and overall survival could be observed in recent years [1]. In the overall patient population, adjuvant chemotherapy (CTX) shows a significant therapeutic benefit [2]. However, focusing on the population of patients with hormone receptor (HR) positive disease, it becomes clear that there are patient subgroups that will be cured just by adjuvant endocrine therapy without adjuvant CTX. Validated prognostic markers are required to identify this subgroup. Recognized conventional prognostic markers with proven relevance are tumor size, nodal status, grading, histological subtype and age. Still, these biomarkers are insufficient to obtain adequate risk stratification in a HR-positive, HER2-negative population. Therefore, a number of additional new molecular biomarkers has been developed and validated which allow a better defined distinction between "high" and "low" risk of metastases.

The urokinase plasminogen activator (uPA) system plays an important role in the process of tumor cell invasion and metastasis [3]. As early as 1988, it was demonstrated that elevated levels of uPA in tumor tissue of breast cancer patients are associated with poor prognosis [4]. Shortly after, it was shown for the first time that also high concentrations of plasminogen activator inhibitor-1 (PAI-1) are associated with a worse clinical course [5]. Observations in various animal models showed that uPA is a crucial factor in the process of tumor progression [6]. uPA and PAI-1 have been investigated as prognostic factors in early breast cancer in many retrospective and prospective, independent clinical trials ever since. The determination of uPA and PAI-1 concentration in tumor tissue by enzyme-linked immunosorbent assay (ELISA) is the first prospectively validated biomarker in early breast cancer with level of evidence (LOE) 1A, following the systematics of Hayes et al [7]. As part of the multicenter Chemo-N0-trial (n = 647, 12 centers), it was shown that in N0 patients the uPA/PAI-1 test can identify a group of patients that can be spared adjuvant CTX [8]. Based on these results and on the data from a comprehensive meta-analysis (n = 8377) conducted by the European Organisation for Research and Treatment of Cancer (EORTC) [9], uPA/PAI-1 has been recommended as biomarker for risk stratification and therapeutic decision making in node-negative breast cancer in the guidelines of the American Society of Clinical Oncology (ASCO) since 2007 as well as of the German Society of Gynecological Oncology (AGO) since 2006 [10] [11]. Despite convincing data and a commercially available ELISA assay for uPA/PAI-1, the proteolytic factors are not generally used as biomarkers for prognostic assessment of early breast cancer in clinical practice. Also uPA/PAI-1 has never been included as a prognostic factor in the recommendations of the St. Gallen Consensus Conference. One reason for this may be that uPA/PAI-1 determination is logistically demanding since the test can be performed only on fresh tissue [12].

Risk stratification based on gene expression analysis was described for the first time by Perou and colleagues with the introduction of the intrinsic subtypes in the molecular pathology of breast cancer [13]. It was shown that various intrinsic subtypes are associated with a different prognosis of the disease, which affects the overall and relapse-free survival [14]. Based on these findings, several commercially available gene expression tests have been developed. The tests allow identifying a group of patients whose prognosis under adjuvant endocrine therapy is so good that adjuvant CTX can be abandoned. For these patients the expected benefits of CTX would be smaller than the risks conferred by CTX. A number of these multiparameter assays has been added to the therapy recommendation of the St. Gallen International Expert Consensus, including the EndoPredict ^®^ test (EPclin) [15].

EPclin is a RNA-based 11-gene expression test which can be carried out on formalin-fixed, paraffin-embedded (FFPE) tumor tissue. It allows the prediction of the likelihood of distant metastases in patients with ER-positive, HER2-negative breast cancer. The test has been validated in four large randomized phase III studies (ABCSG-6: n = 378; ABCSG-8: n = 1324; GEICAM9906: n = 555 and ATAC: n = 928) and thus has the evidence level LOE IB according to Simon et al [7]. EPclin was the first gene expression test to demonstrate that it provides additional prognostic information on top of grading, tumor size, nodal status and ki67 in a cohort of endocrine-sensitive breast cancer patients [16]. More recent data show that EPclin also predicts late metastasis and local recurrence in hormone receptor-positive breast cancer [17,18].

The aim of this prospective study was to compare the two prognostic tests uPA/PAI-1 and EPclin with regard to feasibility, risk stratification, and impact of the test result on adjuvant therapy recommendation in hormone-sensitive, HER2 negative breast cancer with 0–3 positive lymph nodes.

## Materials and methods

395 consecutive female patients with primary invasive estrogen receptor (ER) positive, HER2 negative, intermediate risk (as defined by the Interdisciplinary S3-Guidelines for the Diagnosis, Therapy and Follow-up Care of Breast Cancer [19]) breast cancer were enrolled in the study. All patients underwent primary therapy at the interdisciplinary breast center of Klinikum rechts der Isar, Technische Universität München, Munich, Germany.

Regarding treatment recommendations case discussions were held during an interdisciplinary tumor conference for all patients. The results of the uPA/PAI-1 test and the EPclin were known to the tumor conference. Additionally a second and third conference was held: One conference accounted for all patient information but the EPclin and uPA/ PAI-1 results, another conference was blinded to the EPclin result but was aware of uPA/PAI-1 results. Consequently three separate treatment recommendations were generated.

### Assessment of the EPclin score

The EPclin score is a second generation genomic expression assay calculating risk of recurrence in primary HR positive, HER2 negative breast cancer. A thorough report of the key facts of the EPclin score is reported elsewhere [16]. In sum, the EndoPredict assay (Sividon Diagnostics GmbH, Cologne, Germany) analyses the expression levels of eight genes of interest (BIRC5, UBE2C, DHCR7, RBBP8, IL6ST, AZGP1, MGP and STC2), as well as three normalization genes (CALM2, OAZ1, RPL37A). For the risk score calculation RNA levels assessed by quantitative reverse transcription PCR (RT-qPCR) in FFPE tumor tissue were used. As published previously the PCR results can be translated into a quantitative risk score using a web-based implementation of the EndoPredict algorithm [20]. The RNA score is finally combined with the clinicopathological parameters tumor size and lymph node involvement which leads to the final EPclin test result. The software for the required computations is now CE-marked and available under: https://www2.endopredict.com/EPReportGenerator. The validated cut-offs for EndoPredict score and EPclin score for discrimination into low and high risk of distant recurrence are 5 and 3.3 respectively [16].

### Assessment of uPA and PAI-1 levels

Directly after surgery the tumor tissue was transferred to the pathologist laboratory for further processing into tumor extracts as described previously [21]. In brief, in order to determine uPA and PAI-1 levels Femtelle ELISA assay (Sekisui Diagnostics LLC, formerly American Diagnostic Inc., Stamford, CT, USA) and Pierce protein assay (Rockford, IL) were used. The validated cutoff points were utilized: patients with low concentrations of uPA (< 3 ng/mg of protein) and PAI-1 (<14 ng/mg of protein) were categorized as low risk; pateints with high levels of uPA (≥ 3 ng/mg of protein) or/and PAI-1 (≥14 ng/mg of protein) were categorized as high risk.

### Statistics

The distribution of quantitative and qualitative data is presented by descriptive statistics such as median (range) and absolute (relative) frequencies, respectively. Corresponding hypothesis testing for group comparisons was performed by Mann-Whitney-U tests and Fisher’s exact test. Relations and concordance of histologic parameters as well as EPclin and uPA/PAI-1 values and test results were assessed by Spearman’s rank correlation coefficient and Cohen’s Kappa. Disagreements of classifications were investigated by McNemar’s tests / exact binomial tests. All statistical tests were conducted on exploratory, two-sided 5% significance levels using R 3.2.0 [22].

### Ethics statement

This observational study was approved by the ethics committee of the faculty of medicine at Klinikum rechts der Isar, Technische Universität München. The need for informed consent was waived.

## Results

### Study population

A total of 395 patients were enrolled between March 2012 and March 2015. The median age of the patients was 59 (29–88) years. Exact tumor characteristics are listed in Table 1.

**Table 1:** Tumor characteristics.

Characteristic	number of pt (n = 395)	%
**Tumor size**		
pT1a	22	5,6
pT1b	69	17.5
pT1c	152	38,5
pT2	136	34,4
pT3	16	4
**Tumor subtype**		
ductal	279	70,6
lobular	74	18,7
ductulo-lobular	19	4,8
tubular	16	4,1
mucinous	4	1
papillary	1	0,3
medullary	2	0,5
**Grading**		
G1	80	20,3
G2	255	64,6
G3	60	15,1
**Nodal status**		
pN0	304	77
pN+ (mi)	14	3,5
pN+ (1–3)	77	19,5

### Test results EPclin and uPA/PAI-1

**Fig 1:**
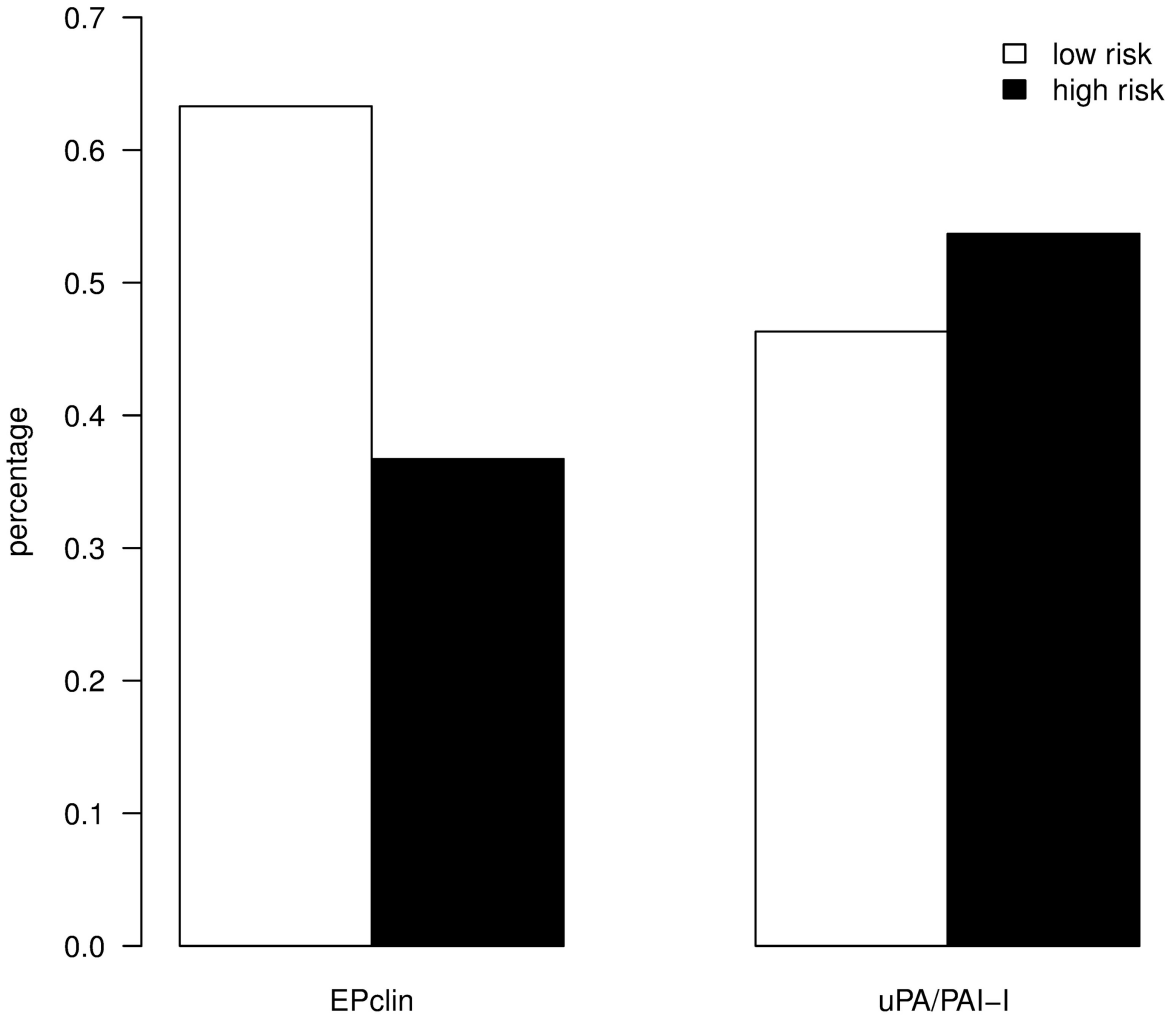
Distribution of risk classes based on EPclin and uPA/PAI-1 test results.

The EPclin test was carried out on all 395 tumor samples. The test result allocated 250 patients (63%) in the low-risk group and 145 patients (37%) in the high-risk group. uPA/PAI-1 was evaluated in 190 (48%) of the tumor samples. The uPA/PAI-1 test allocated 88 patients (46%) in the low-risk group and 102 patients (54%) in the high-risk group (Fig 1).

### Correlation between test results and histological grading

**Fig 2:**
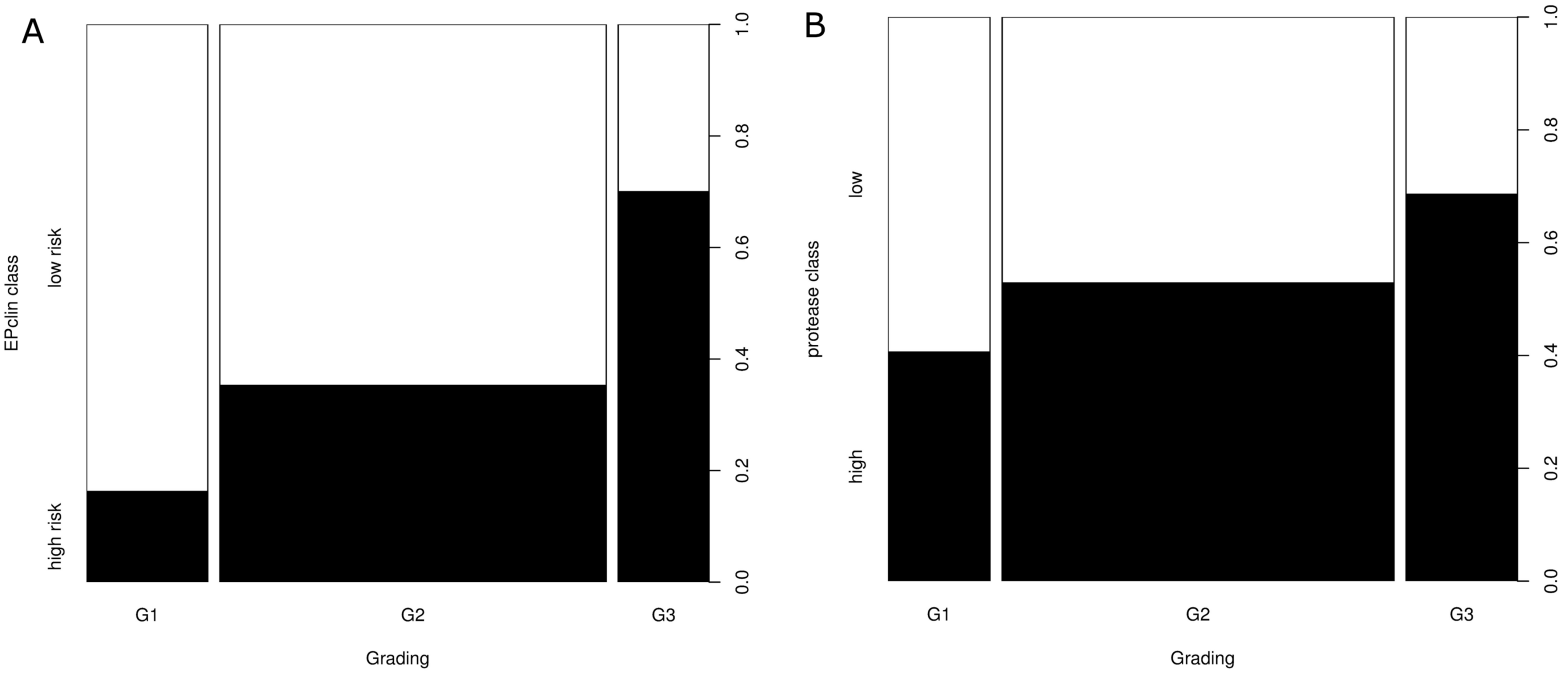
EPclin shows a stronger correlation with grading than uPA/PAI-1. (A) Distribution of the EPclin class as a function of histopathological parameter of grading. Spearman's correlation rho = 0.32; p<0.001. The width of the bars represents the number of observations. (B) Distribution of the protease class as a function of histopathological parameter of grading. Spearman's correlation rho = 0.17; p = 0.021. The width of the bars represents the number of observations.

Fig 2 shows the distribution of the test results as a function of conventional histopathological parameter of grading. Both tests show a significant but weak correlation, whereby the EPclin class (Fig 2A) shows a stronger correlation with grading than the protease class (Fig 2B) (Spearman's correlation rho = 0.32; p <0.001 vs. rho = 0.17; p = 0.021).

### Correlation between EPclin score and level of uPA/PAI-1

**Fig 3:**
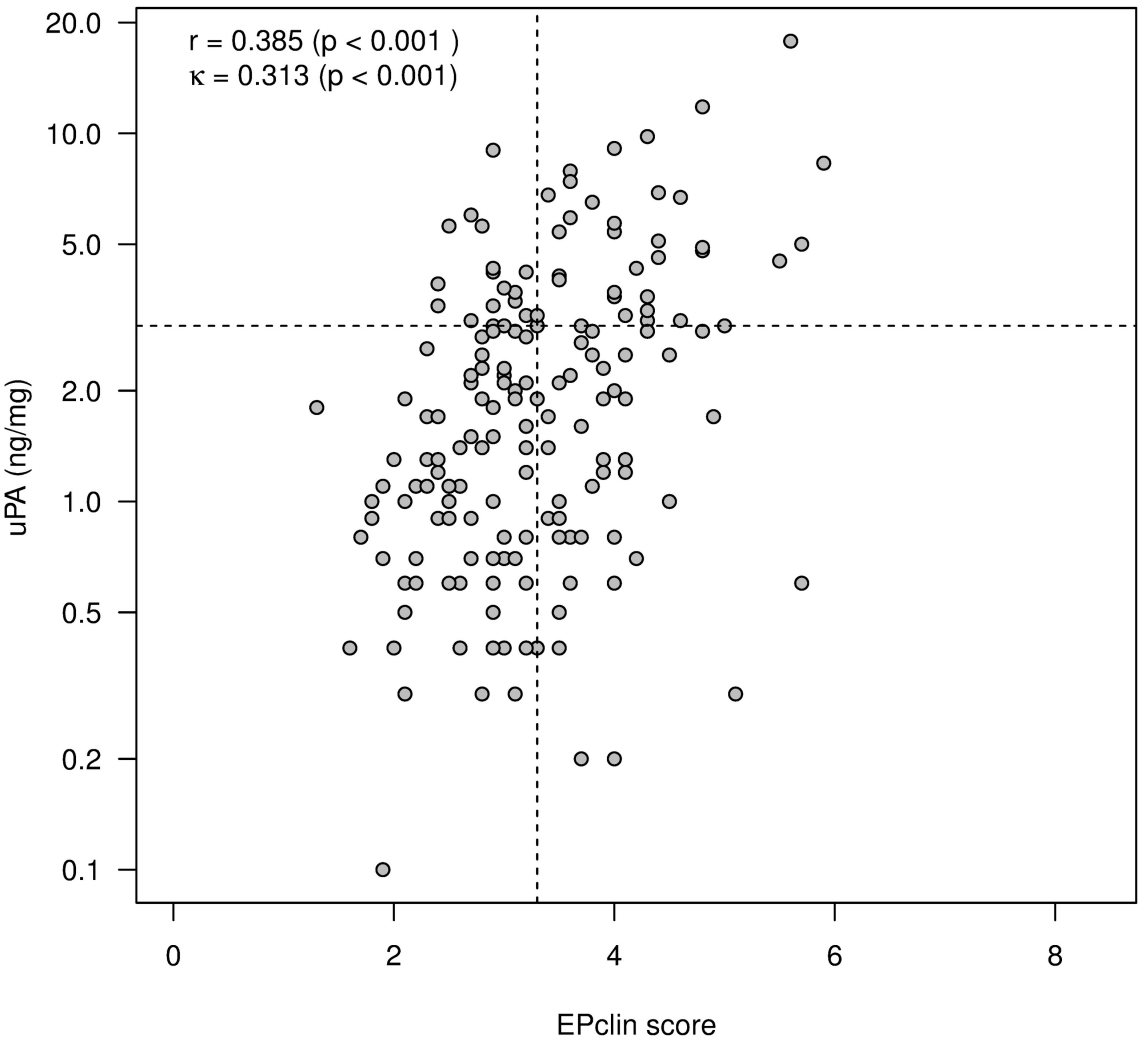
Moderate correlation between EPclin and uPA. Relations are quantified by Spearman’s rank correlation coefficient (r). Allocation to risk classes is indicated by dashed lines. Corresponding concordance is measured by Cohen's kappa (κ).

Fig 3 shows that comparison of EPclin scores with corresponding u-PA values results in a moderate correlation between EPclin and uPA.

**Fig 4:**
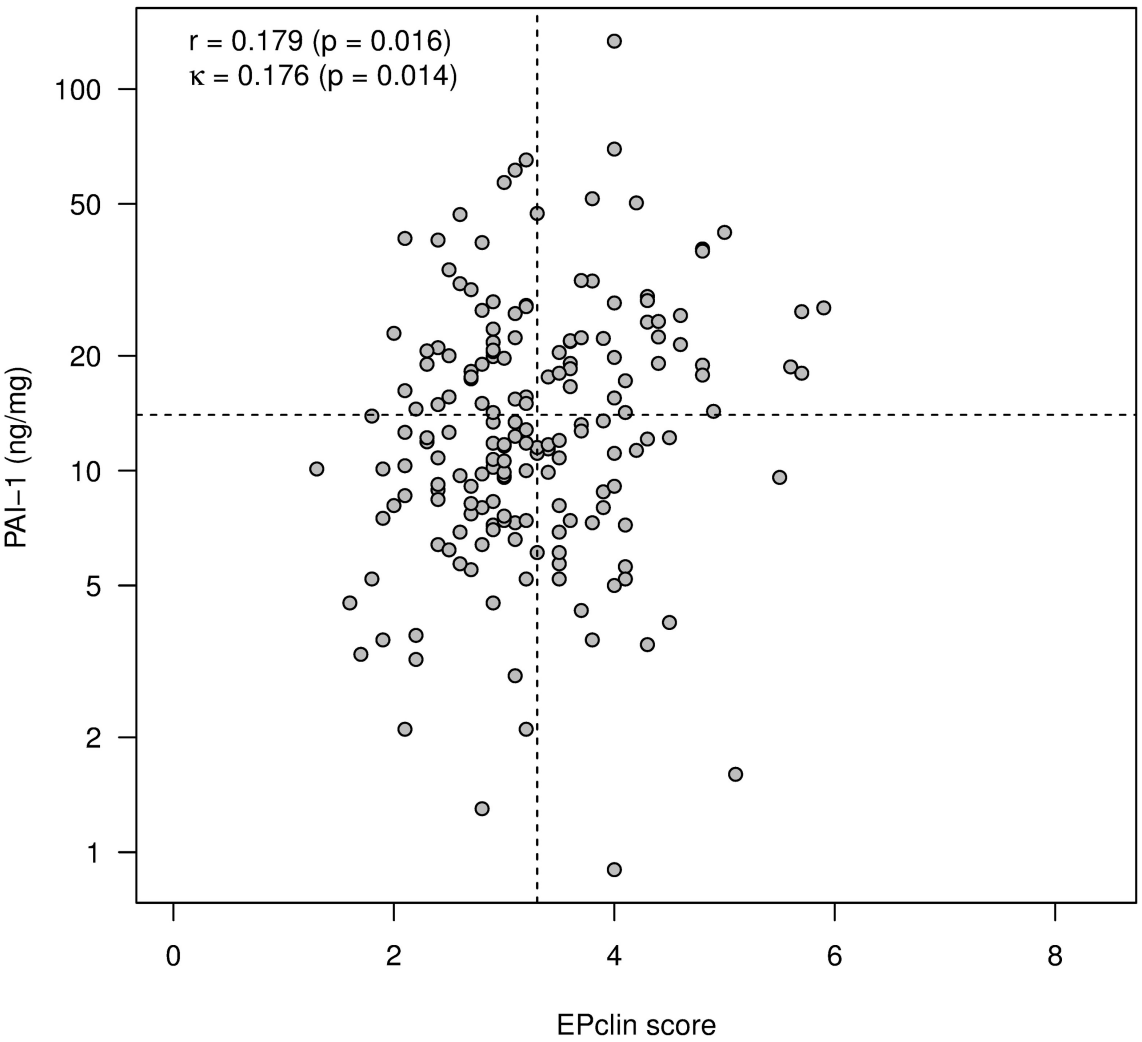
Very weak correlation between EPclin and PAI-1. Relations are quantified by Spearman’s rank correlation coefficient (r). Allocation to risk classes is indicated by dashed lines. Corresponding concordance is measured by Cohen's kappa (κ).

In contrast, only very weak correlation exists between EPclin and PAI-1 (Fig 4).

Moreover, a dichotomization based on established threshold values (EPclin values: 3.3, uPA: 3, PAI-1: 14) shows a moderate or weak concordance of the classification into low-risk and high-risk groups (Figs 3 and 4; Cohens´s kappa κ = 0.313; p <0.001 vs. κ = 0.176; p = 0.066).

### Comparison of risk classification by EPclin and uPA/PAI-1

In 190 cases of the whole study population both tests could be performed. Table 2 shows comparison between risk classification by EPclin and by uPA/PAI-1.

**Table 2:** Risk classification by EPclin vs. uPA/PAI-1.

n = 190	uPA/PAI-1 high risk	uPA/PAI-1 low risk
EPclin high risk	52 (27%)	27 (15%)
EPclin low risk	50 (26%)	61 (32%)

In 59% of cases, both tests resulted in allocation into the same group. A dissimilar risk allocation resulted in 41% of cases, which tended to be more frequent in the group with high uPA/PAI-1 levels (26%) (McNemar's test, p = 0.009).

### Comparison of decision impact by EPclin and uPA/PAI-1

Table 3 shows the frequencies of change in therapy recommendation (decision impact) by the uPA/PAI-1 results set against the decision impact by the EPclin test.

**Table 3:** Decision impact by EPclin vs. uPA/PAI-1.

n = 190	no impact according to uPA/PAI-1	minus CTX according to uPA/PAI-1	plus CTX according to uPA/PAI-1
no impact according to EPclin	90 (47%)	10 (5%)	3 (2%)
minus CTX according to EPclin	53 (28%)	32 (17%)	0
plus CTX according to EPclin	1 (0.5%)	0	1 (0.5%)

The recommendations coincide in 123 (65%) of 190 cases. They differ in 67 (35%) of 190 cases, whereby at different results, the therapy recommendation is significantly more frequently affected by EPclin than by uPA/PAI-1, namely in 54 (28%) cases versus 13 (7%) cases (p<0.001, exact binomial test). In cases of disparate recommendations, CTX was not recommended by uPA/PAI-1 in 10 (77%) of 13 cases and by EPclin in 53 (98%) of 54 cases (p = 0.021, Fisher's exact test).

**Fig 5:**
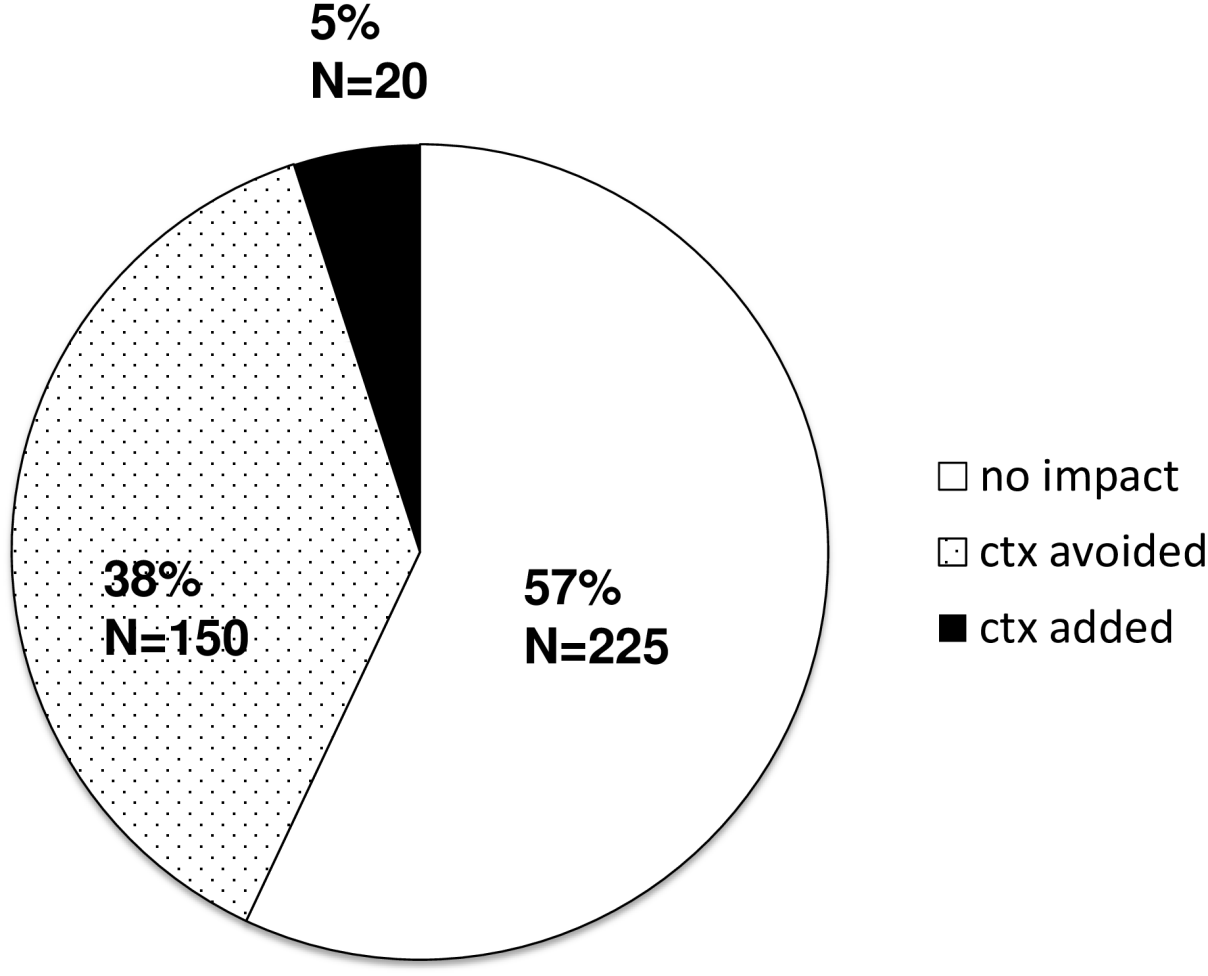
Decision impact by EPclin in the overall study population. Interdisciplinary tumor conference was aware of both EPclin and uPA/PAI-1 results.

Fig 5 shows decision impact of EPclin test results in the overall population of 395 patients.

With results of the uPA/PAI-1 test on 190 patients taken into account, the EPclin test resulted in a change in therapy recommendation in 170 patients (41%), favoring avoidance of CTX 150 times (38%) and its addition 20 times (5%).

**Fig 6:**
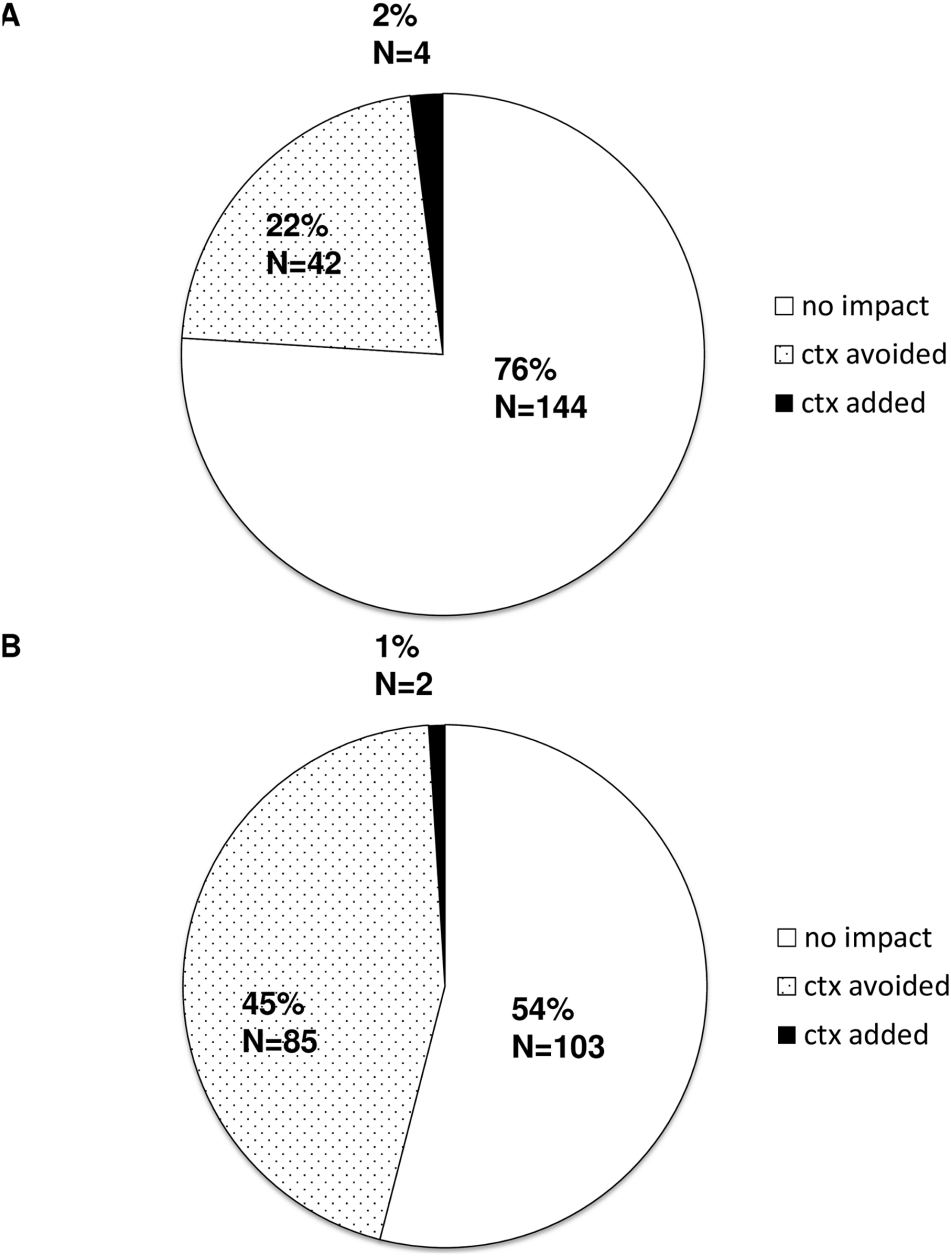
Decision impact by EPclin is stronger compared to decision impact by uPA/PAI-1. (A) Decision impact by uPA/PAI-1. (B) Decision impact by EPclin.

Fig 6 refers only to the 190 patients in whom both the EPclin test and uPA/PAI-1 test were carried out, thus allowing direct comparison of the decision impact of EPclin and uPA/PAI-1.

The uPA/PAI-1 test resulted in a change in therapy recommendation in 46 patients (24%), favoring avoidance of CTX 42 times (22%) and its addition 4 times (2%) (Fig 6A). In contrast, the EPclin test resulted in a change in therapy recommendation in 87 patients (46%), favoring avoidance of CTX 85 times (45%) and its addition 2 times (1%) (Fig 6B).

## Discussion

In this study, two standardized and validated prognostic tests—EPclin and uPA/PAI-1- were prospectively compared for the first time with respect to feasibility, risk stratification and influence on adjuvant therapy recommendation in endocrine sensitive, HER2- negative early breast cancer.

EPclin resulted significantly more frequently in a change of therapy recommendation than the uPA/PAI-1 test (46% vs. 24%). The change was in almost all cases abstention from recommendation of adjuvant CTX (45% vs. 22%) so that recommendation of adjuvant CTX was abandoned twice as often by EPclin compared to the uPA/PAI-1 test. EPclin is therefore clearly superior to the uPA/PAI-1 test with respect to possible avoidance of adjuvant CTX. This is of paramount importance in clinical practice as implementation of EPclin could reduce overtreatment and associated CTX-induced toxicities in the individual patient to a greater extent than uPA/PAI-1. Moreover substantial treatment-related healthcare costs could be further reduced. Blank et al analyzed the health economic effect of EPclin in the population of 1619 patients of the ABCSG 6/8 trials and determined that EPclin-based risk stratification presents a cost-effective tool for reducing CTX-associated costs [23].

EPclin and uPA/PAI-1 have been validated for risk stratification of patients with endocrine sensitive early breast cancer and have been used already for some time in clinical routine. The risk stratification performed in the context of the present study by uPA/PAI-1 (46% low risk, 54% high risk) and EPclin (63% low risk, 37% high risk) is consistent with published data from other retrospective, monocentric surveys; recently, Kolben et al reported about 381 intermediate-risk patients, of which 45% were uPA/PAI-1 low risk and 55% were uPA/PAI-1 high risk [24]. Müller et al report a EPclin low-risk group of 48% in a cohort of 167 defined tumors in which smaller tumors (pT1a/b) were under-represented compared to the present study [20]. Schlake et al report about a cohort of 82 patients in which EPclin lead to a low-risk classification in 68% of the cases [25].

Our study provides for the first time data for direct comparison of risk stratification by uPA/PAI-1 and EPclin: In 77 (41%) of 190 cases in which both tests were performed, the results were discrepant, i.e., different risk allocations resulted depending on the used test result. The discrepancy and the only moderate correlation between the two tests can be explained by the different test characteristics: EPclin determines expression levels of genes associated with tumor cell proliferation and hormone receptor activity at the RNA level. The uPA/PAI-1 assay determines the uPA and PAI-1 content of tumor tissue at the protein level. UPA and PAI-1 are responsible for pericellular mechanisms such as localized proteolysis, cell adhesion and migration [26]. Unlike PAI-1, uPA has been attributed with a proliferation-promoting role, similar to that of a growth factor [27]. This may explain why we could demonstrate a moderate correlation between EP and uPA values (r = 0.385) while EPclin and PAI-1 values correlated only very weakly (r = 0.179). The measured differences in the strength of correlation between EPclin or uPA/PAI-1 and the conventional histopathological prognostic parameter of grading also suggest that the two tests map distinct biological properties of the tumor. It is also likely that inclusion of the clinicopathological parameters tumor size and nodal status provides additional prognostic information to the EPclin score, which is disregarded in the UPA/PA-1 test also contributing to divergent results.

This is not the first time that use of two different prognostic tests on a defined cohort has shown to produce different results in one and the same patient. Even comparison of two different gene expression tests leads to discrepant test results: Varga et al retrospectively determined the EPclin score in 34 patients for whom an Oncotype DX recurrence score was available. With 76%, only a moderate concordance of the two gene expression tests was demonstrated. The authors attribute this to the different weighting of the various biological properties of the tumor measured at the gene expression level [28].

EPclin was clearly superior to uPA/PAI-1 with regard to feasibility in clinical practice: EPclin could be determined in all 395 tumor samples included in the study. In contrast, uPA/PAI-1 test was performed in only 190 (48%) of the tumor samples. Similar data on limited feasibility of the uPA/PAI-1 test can be found in a French study in which the uPA/PAI-1 test was feasible in only 57% of a total of 285 tumors [29]. In a retrospective study by Müller et al, test feasibility for EPclin was 99%, which is similar to that in our study [20]. The difference in test feasibility in our study is due to the fact that the uPA/PAI-1 test can be performed exclusively on fresh tissue. Tissue for the uPA/PAI-1 test must be collected immediately after surgery as a frozen section. About 50 mg of tumor tissue is required for protein determination, which makes the test often not feasible for small tumors. Furthermore, fresh tissue collection in clinical routine is far more demanding than collection of FFPE tissue samples. In contrast to EPclin, postoperative determination of uPA/PAI-1 in formalin- FFPE tissue is not established for clinical routine yet. EPclin can be determined from a single 5 μm FFPE tissue section.

The novelty of this study is that two different prognostic tests were used prospectively in clinical routine, and that both test results and therapy recommendations were compared with each other. Thus, it could be shown that the existing discrepancy in the test results indeed leads to a significant change in the recommendation of adjuvant therapy. Use of the EPclin test is significantly more likely to result in refraining from adjuvant CTX. Limitations of this study include the unicentric and non-randomized design and the lack of data on the relapse free survival of the patients. It is of paramount clinical relevance to determine the effect of avoidance of CTX on the prognosis of this patient population. Data on disease-free and metastasis-free survival is being collected prospectively in the course of a follow-up study and will be available in near future.

## Conclusion

In summary, our study shows that EPclin is superior to uPA/PAI-1 with respect to feasibility and impact on recommendation of adjuvant therapy. Unlike the logistically more demanding uPA/PA-1, EPclin is feasible in routine clinical practice and results in substantial avoidance of adjuvant CTX in endocrine-sensitive, HER2-negative breast cancer. Prospective evaluation of distant relapse free survival of patients for whom therapy decision has been influenced by EPclin is ongoing.
